# Burns: Their Immediate Local Treatment

**Published:** 1907-05-18

**Authors:** 


### Points in Surgery.

### BURNS: Their Immediate Local Treatment.

In cases of burns death may be due first to asphyxia, secondly to shock, and thirdly to septicæmia. The medical man seldom gets to the case in time to treat the first condition; the second is essentially a general condition; while the whole success in preventing the third depends upon the immediate local treatment. It is, therefore, the condition which must be considered here.

### A Mistaken Idea.

Among the public it is a generally accepted idea that the thing to do in the case of a burn is to dust flour over it or to cover it with oil, and, indeed, even in some comparatively late text-books on surgery, a mixture known as "Carron Oil" is advocated. The use of such applications cannot be too strongly deprecated, and, indeed, if the lay mind could be taught that the best thing to put on a burn before the doctor is called is a hot compress which should contain some boracic acid if there is any in the house, it is probable that the majority of deaths due to septicæmia after burns would be prevented. For the whole aim and object of the local treatment is to prevent sepsis; flour and olive oil may be very soothing and may allay the pain, but there is no antiseptic property in them; rather they are excellent culture media for bacteria.

### To Prevent Sepsis.

To repeat then—and the fact cannot be too strongly emphasised—the local treatment of burns is essentially to prevent sepsis; this is to be obtained in two ways, firstly by removing anything from the burnt area which may in any way favour sepsis, and secondly by applying some antiseptic dressing.

The form of burn most commonly seen may be taken as an example, namely, one of the second and first degree, where there is a bleb containing clear fluid, and around this a reddened area of skin; and here it must be clearly understood that no burn, however small, is too insignificant to need the most careful attention; when once it has been allowed to go septic, a burnt area the size of the palm of the hand is large enough to generate toxins sufficient to cause death by absorption. In the first cleaning up of the burn the amount of shock accompanying it must be carefully considered. The question to be decided is, how much active treatment may be safely employed without increasing the shock to a degree dangerous to life? What must be aimed at is the aseptic removal of all the loose-lying epidermis and of the fluids beneath. The mere pricking of the blebs should not be considered enough; when this only is done a considerable amount of débris is left beneath, and in this bacteria may readily flourish and may subsequently give rise to sapræmia and septicæmia.

### Cleaning Up a Burn.

If the shock be not too great, to scrub the burn under an anæsthetic is undoubtedly an excellent measure; by this means the whole burnt area can be efficiently cleaned and dressed in a very short space of time. Any mild antiseptic lotion may be used, as warm boracic lotion, perchloride of mercury 1 in 3,000, or carbolic 1 in 200, but certainly the best in this case is warm weak lysol [.25 per cent.]; this is antiseptic, it is not irritating in the above strength, and the alkali in it combines with the fat on the surface of the skin and enables this to be easily removed; it can be applied lightly with a sterilised scrubbing-brush, or with a piece of boiled lint rolled up into a ball. If, however, an anæsthetic is not considered safe, if there are no facilities for giving it, or if the burn be very small, much can be done by removing the epithelium with sterilised forceps and scissors. This process does not hurt the patient if the epithelium be gently picked up and care be taken not to pull on the line of junction between the raised-up epithelium and that which remains attached; the area then may be gently washed over with the lysol lotion. At the same time that the burnt area is cleaned it is advisable to clean the skin around; the hands or feet are particularly liable to be dirty and, if near the burn, to be a source of danger at later dressings.

### Dressing a Burn.

When the cleaning has been done the choice of dressing is a large one. That which is easiest obtained and most generally applicable in the early stages is a hot boracic fomentation, which may be changed every four or six hours. Indeed, in many cases these fomentations are also the best, if there is any chance that the first cleaning is not as thorough as might be desired. This is true, for instance, when the shock is so great that it is not thought wise to expose the patient to a long dressing, or when the burn is near the genitalia, the anus or the mouth, whence it may become infected. On the other hand, if the first cleaning has been thorough and there is no particular factor predisposing to sepsis, a dry dressing is better, for it need not be changed so often and, therefore, the patient need not be so much disturbed. The eucalyptus ointment (B.P.) spread on lint is, perhaps, the best application; it is, however, rather expensive and some people object to the smell. A dressing composed of boric acid gr. xx. mixed in ℥j. of soft paraffin is good, but is hardly antiseptic enough, even for a well-cleaned burn. Picric acid solution (.5 per cent.) may be used, but it has no especial advantage, and its staining properties make it a very difficult dressing to put on without doing damage to sheets and clothes. A very good dressing is a powder consisting of salicylic acid 1 part and boracic acid 5 parts, powdered on and covered by a piece of dry lint; but it is apt to stick and it makes the redressing very painful; also it smarts for some time after it is applied.

In conclusion, the necessity of dressing the burn at once cannot be too strongly insisted on; if the treatment above described be carried out at once septicæmia should not be anticipated even in a very dirty limb, but if the burn be left for 24 hours such an issue is much to be feared.

